# Neuroblastoma response to RAS-MAPK inhibitors and APR-246 (eprenetapopt) co-treatment is dependent on SLC7A11

**DOI:** 10.3389/fonc.2024.1433256

**Published:** 2024-12-06

**Authors:** Vid Mlakar, Ina Oehme, Laurence Lesne, Sara Najafi, Marc Ansari, Fabienne Gumy-Pause

**Affiliations:** ^1^ Cansearch Research Platform for Pediatric Oncology and Hematology, Department of Pediatrics, Gynecology and Obstetrics, Faculty of Medicine, University of Geneva, Geneva, Switzerland; ^2^ Hopp Children’s Cancer Center Heidelberg (KiTZ), Heidelberg, Germany; ^3^ Clinical Cooperation Unit Pediatric Oncology, German Cancer Research Center (DKFZ) and German Cancer Consortium (DKTK), Heidelberg, Germany; ^4^ Department of Pediatric Oncology, Hematology and Immunology, University Hospital Heidelberg, Heidelberg, Germany; ^5^ Division of Pediatric Oncology and Hematology, Department of Women, Child and Adolescent, University Geneva Hospitals, Geneva, Switzerland

**Keywords:** neuroblastoma, APR-246, ERK1/2, RAS, MAPK, SLC7A11, Hsp27, PRIMA-1 Met

## Abstract

**Background:**

We previously demonstrated that APR-246 (eprenetapopt) could be an efficient treatment option against neuroblastoma (NB), the most common pediatric extracranial solid tumor. APR-246’s mechanism of action is not completely understood and can differ between cell types. Here we investigate the involvement of well-known oncogenic pathways in NB’s response to APR-246.

**Methods:**

A proteome profiler kinase assays and western blot analysis were used to identify the molecular pathways involved in the responses to APR-246. Bulk ATP levels were used to determine the viability of cells and the IC_50_ for APR-246. Cystine-FITC was used to measure the cellular uptake of cysteine. PmRNA5 was used to activate ERK1/2 and pshRNA1 was used to silence HSP27. An IMR-32 xenograft zebrafish embryo model was used to assess APR-246 and sulfasalazine efficacy *in vivo*.

**Results:**

After APR-246 treatment, the most deregulated signaling protein identified was ERK1/2, an end-point kinase of the RAS-MAPK pathway. Induction of phospho-ERK1/2 resulted in increased glutathione (GSH) levels, increased cystine uptake, and increased resistance of NB cells to APR-246. Using ERK1/2 inhibitors in combination with APR-246, we were able to categorize cells into synergistic and antagonistic groups. After co-treatment, these two groups differ by their levels of SLC7A11 and Hsp27 phosphorylation, cystine uptake, and BIM expression. Using erastin and sulfasalazine, both inhibitors of SLC7A11 and activators of ferroptosis, we were able to reverse the antagonistic effects of ERK1/2 inhibitors and demonstrate a strong synergistic action *in vitro* and *in vivo* in zebrafish models.

**Conclusions:**

These results demonstrated a pivotal role of the RAS-MAPK pathway in the NB cellular response to APR-246 via the modulation of intracellular concentrations of GSH and the transport of cystine through SLC7A11, phosphorylation of Hsp27, and programmed cell death. Combining APR-246 with RAS-MAPK pathway inhibitors can, in some cases, lead to antagonistic action, which can be reversed by combining APR-246 with the clinically approved drug sulfasalazine.

## Background

Neuroblastoma (NB) is the most common pediatric extracranial solid tumor. Current event-free survival rates range from 75% to more than 85% for low- and very low-risk groups, to less than 50% for high-risk (HR) patients, despite dose-intensive therapy (1, 2). HR NBs are characterized by specific molecular events, the most common of which are *MYCN* amplification (MNA) (2, 3) and 11q deletion (4). NB has been proposed as a good candidate for p53 pathway reactivation because it has a low frequency of mutations in *TP53* (5, 6). In addition, the downstream pathway is usually intact, with most of the mutations appearing to be in the upstream MDM2-p14(ARF)-p53 network (7).

Our group and others recently demonstrated that APR-246 could be an efficient treatment against NB (8, 9). Although APR-246 is described as a p53 reactivator (10, 11), it was demonstrated that in NB p53 likely plays limited and indirect role by the modulation of glutathione (GSH) metabolism (8, 9) via SLC7A11 (12, 13). Involvement of other pathways could also be important in NB (9). The present study is not the first to have questioned p53’s direct role in APR-246-induced cell death. Many reports have provided evidence of APR-246’s efficacy in p53-null cancers and of the various modalities of cell death it causes (14–18). An extensive literature review suggested that the true cause of cell death due to exposure to APR-246 remains elusive and may not be ubiquitous but dependent on cell type (16, 18). The best working hypothesis so far appears to be that APR-246 induces generalized oxidative stress and the subsequent activation of apoptosis and/or ferroptosis, where p53 plays a role in the control of GSH levels by inducing the expression of SLC7A11 (12, 19).

Because the modalities of APR-246’s action can vary between cell types, the present study’s goal was to investigate the most common phosphorylation signaling pathways induced by APR-246 in NB. We identified phosphorylation of ERK1/2, the RAS-MAPK pathway endpoint kinase, as an effect of APR-246 treatment and investigated its role in the cellular response to APR-246.

## Methods

### Cell lines and chemicals

CHP212, NBL-S NB cell lines were provided by Dr. E. Attiyeh and Prof. J. Maris (Children’s Hospital of Philadelphia, Philadelphia, USA). BE-(2)C, IMR-32, LA1-55N, KCNR, and SK-N-DZ cell lines were purchased from ATCC (USA). The characteristics of cell lines are shown in **Table 1**. All these cell lines were maintained in a standard NB medium of DMEM supplemented with 10% FBS, 1% antibiotic/antimycotic solution, and 1% L-glutamine. Each line underwent independent identity and mycoplasma testing performed by Microsynth AG (Switzerland). The following compounds were used: alectinib, APR-246 (50 mM in H_2_O, Abcam, UK), cystine-FITC, erastin, ferrostatin-1, SCH772984, sulfasalazine (SSZ), Z-VAD-FMK. Stably expressed caMEK (NBL-S, SK-N-DZ, SK-N-SH) and dnMEK (BE-2C) were obtained by the transfection of pmRNA5, containing the caMEK or dnMEK variant of MEK1, respectively. The stable knock-down of Hsp27 was obtained by the transfection of the following shRNA: shRNA1 - 5’-CGCCCCGGACGAGCTGACGGTCAATTCAAGAGATTGACCGTCAGCTCGTCCGGGGCG-3’ and shRNA2 – 5’-CCGATGAGACTGCCGCCAAGTTTCAAGAGAACTTGGCGGCAGTCTCATCGG-3’ in the pshRNA1 vector. All transfections were performed according to the manufacturer’s recommendations using the X-tremeGENE HP DNA Transfection Reagent (Roche, Switzerland). The selection was performed using blasticidin (Merck Millipore, USA). Vehicle controls, empty vectors, or scrambled controls were used in all the experiments, as indicated.

**Table 1:** Characteristics of the neuroblastoma cell lines used in this study.

Cell line	MYCN	TP53 status and p53 expression	ALK status	Ref
BE-2C	amp	C135F High expression	wt	(8)
CHP212	amp	wt	wt	(8)
IMR32	amp	wt	wt	(36, 37)
KCNR	amp	wt	F1174L	(36, 38)
LA1-55N	amp	No expression	F1174L	(8)
NBL-S	wt	wt	wt	(8)
SK-N-DZ	amp	R110L (rs11540654) High expression	wt	(8)
SK-N-SH	wt	wt	F1174L	(8)

Amp, amplification; Wt, wild-type.

### Cell survival assay

Cell viability was measured using the CellTiter 2.0 (Promega, USA) ATP-based assay according to the manufacturer’s recommendations for measuring the number of live cells. 50 μL of CellTiter 2.0 was added to each well containing 10,000 previously treated cells, as indicated in the figures. Plates were incubated for 10 min on a horizontal shaker at medium speed and room temperature (RT). Chemiluminescence was measured using a SpectraMax iD3 (Molecular Devices, USA) at RT.

### Proteome profiler assay and western blots

A proteome profiler assay was performed on the SK-N-DZ cell line treated with 60 μM APR-246 for 30 min and 6 h, and on the BE-2C (5 μM), CHP212 (0.5 μM), NBL-S (10 μM), LA1-55N (10 μM), KCNR (10 μM) and SK-N-DZ (10 μM) cell lines treated with SCH772984 for 12 h. After treatment, cells were collected by scraping and processed according to the manufacturer’s recommendations. Images were captured using an iBlot device (Invitrogen, USA) and analyzed using Image Studio Lite Ver. 5.2 software (Li-Cor, Lincoln, Nebraska, USA).

The following primary antibodies were used for western blots. AKT123 (ab179463), phospho-AKT123 (ab192623), BCLXL (ab32370), BCL-2 (ab182858), beta-actin (ab6276), Hsp27 (ab2790), Hsp27-phospho-S78 (ab32501), JNK (ab199380), phospho-JNK (ab215208), MEK1/2 (ab178876), p53-phospho-Ser15 (ab223868), p53-phospho-Ser46 (ab76242), p53-phospho-Ser392 (ab33889), SLC7A11 (ab307601), vinculine (ab129002) from Abcam (UK). ERK1/2 (MA-15134), ERK1/2-phospho-T185-T187 (700012) and MEK1-phospho-S218- S222 (MA5-32165) from ThermoFisher. BAX (5023P), BIM (2933), BIM-phospho-S69 (4581) from CellSignaling. MYCN (10159-2-AP) from ProteinTech. p53 (sc-126) from SantaCruz.

### Total glutathione and cystine transport measurement

The concentration of total intracellular glutathione (GSH-Glo Glutathione Assay, Promega, USA) was measured according to the manufacturer’s recommendations. Chemiluminescence was measured using a SpectraMax iD3 device (Molecular Devices, USA) at RT. Cystine transport was measured using cystine-FITC and fluorescence-activated cell sorting (FACS). 250,000 cells were plated in six-well plates, treated and incubated in Hank’s balanced salt solution (HBSS) for 45 min. Next, HBSS was replaced with a medium supplemented with cystine-FITC (5 uM), and cells were incubated for 5, 10, and 20 min. The reaction was stopped by incubating the cells on ice. Cells were washed once in ice-cold PBS, resuspended in 300 μL of HBSS, and analyzed using a Cytoflex flow cytometer (Beckman Coulter, USA). 1 μL of DRAQ7 was used to label the dead cells. The fluorescence of intracellular cystine-FITC was measured using 543 nm and 653 nm filters. The transporter’s activity was calculated by plotting the average fluorescence of 10,000 cells at 5, 10, and 20 min and liner fitting of the points. The slope of the line served as a measurement of the Cystine-FITC transport activity. All experiments were replicated at least three times.

### Ferroptosis measurement

Invitrogen™ Image-iT™ Lipid Peroxidation Kit (Invitrogen, USA) kit was used to measure the creation of lipid peroxidation, a main marker of ferroptosis cell death, according to the manufacturer’s recommendations. Briefly, 250,000 NBLS-S or KCNR cells were exposed to 60 μM APR-246 for 6 hours. Induction of lipid peroxides was measured at 488 nm wavelength using a Cytoflex flow cytometer (Beckman Coulter, USA).

### Zebrafish *in vivo* assay

Wildtype zebrafish embryos of the AB line were used for xenograft experiments. The fish were cared for and bred under standard conditions, as described previously (20). Briefly, fish were raised and bred at 28°C and for the experiments, eggs were collected and placed in E3 embryonic buffer. 24 h post-fertilization (hpf) 0.2 mM 1-phenyl-2-thiourea (PTU, Sigma-Aldrich, Merck KGaA, Darmstadt, Germany) was added.

The toxicity of APR-246 and SSZ on the embryos was tested before the start of the xenograft experiments. For this, embryos were exposed to the compounds at 48 hpf and were imaged with a stereo microscope (Leica) at 72 hpf and 120 hpf. Assessment of toxicity included death, morphological changes (e.g. edema or curvature of body), and behavioral changes.

The xenograft experiments were done as described previously (20, 21). Briefly, approximately 150-250 of fluorescently labeled (CellTracker CM-DiI (#C7000, ThermoFisher Scientific Inc., Waltham, MA, USA)) IMR-32 cells were injected with the FemtoJet express microinjector (Eppendorf, Hamburg, Germany) into the yolk sac of each zebrafish embryo at 48hpfs. After injection, embryos were kept at 34°C. It is generally estimated that zebrafish embryos take up approximately 1/20 to 1/10 of the drug concentration that has been applied (Gatzweiler et al. and references herein). Tumor volume was assessed with a Zeiss LSM 710 confocal microscope (Zeiss, Oberkochen, Germany) 24 h post-injection (hpi), and after having been exposed to treatment for 48 h (120 hpf). Tumor development was rated for each embryo individually by calculating change from baseline. The zebrafish-adapted Response Evaluation Criteria in Solid Tumors (RECIST) as described previously (Wrobel et al) was used to evaluate and visualize the drug response. To be classified as progressive disease (PD) the tumor volume must have increased at least 20%, and to be classified as partial response (PR) the tumor volume must have decreased by more than 30%.

### Statistics

A robust nonlinear fit of dose vs. response method (GraphPad Prism 7, USA) was used to calculate the IC_50_ from relative cell-survival measurements, as determined using CellTiter2.0. A linear fit was used to calculate the speed of cystine-FITC uptake (GraphPad Prism 7, USA). The difference in speed is given by the ratio between treated cells and non-treated controls. The coefficient of drug interaction (CDI) was calculated using the following formula: CDI = AB/(AxB), where AB represents the relative cell viability for the combination of drugs, and A and B represent the relative viability for each compound alone. A CDI less than 0.8 was considered indicative of synergy. A CDI between 0.8 and 1.2 was considered additive, and a CDI greater than 1.2 indicated antagonism (22). A paired two-way t-test was used to calculate statistical significance. Unless otherwise noted, triplicate measurements were taken in each experiment, and at least two independent experiments were performed. The zebrafish-adapted Response Evaluation Criteria in Solid Tumors (RECIST) were used to evaluate and visualize the drug response, as described previously (20). To be classified as a progressive disease (PD) tumor volume had to have increased by at least 20%, and to be classified as partially response (PR) tumor volume had to have decreased by more than 30%.

## Results

### MEK1/2, ERK1/2 phosphorylation is modulated by APR-246

**Figure 1:**
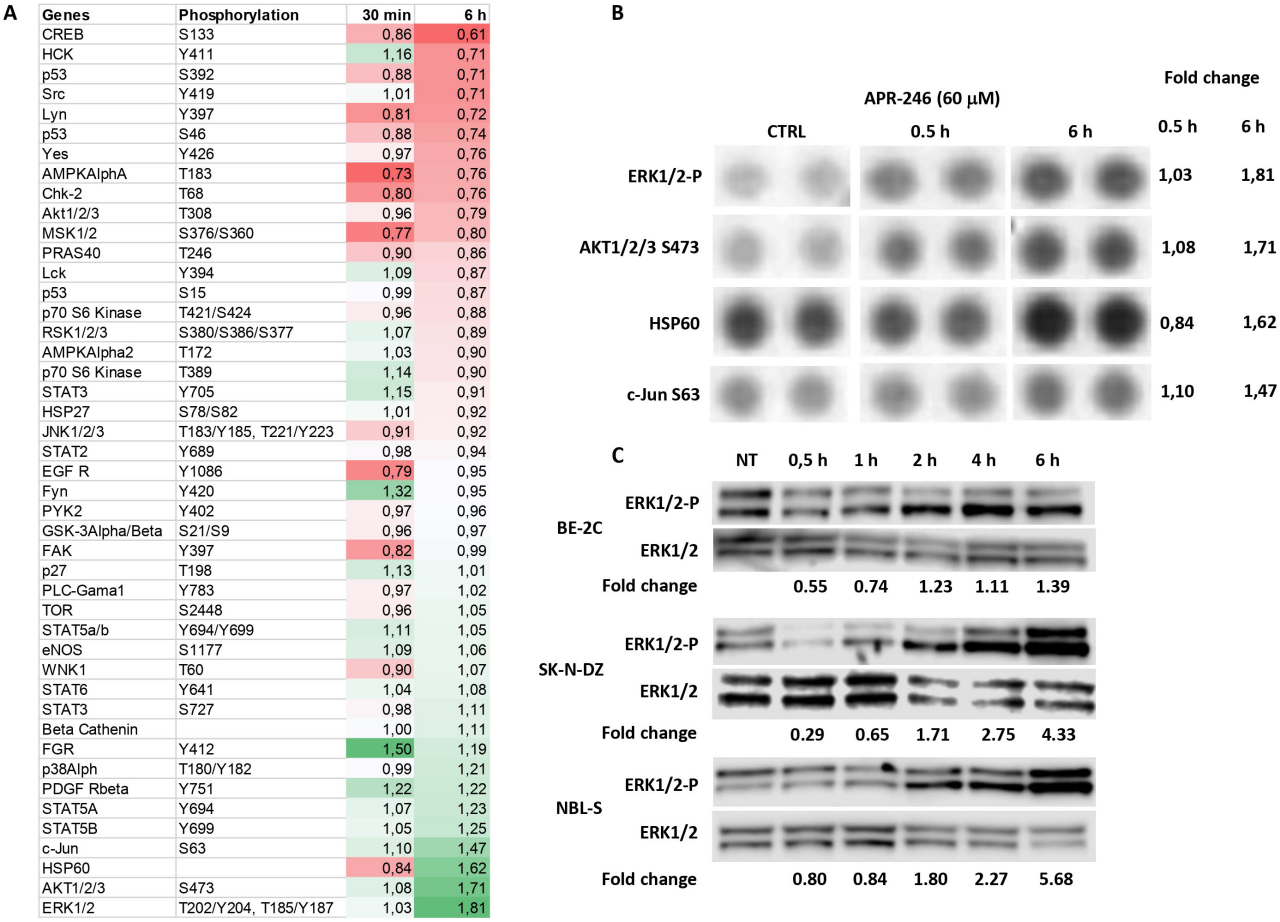
Identification of RAS-MAPK pathway as a target of APR-246. **(A)** Protein phosphorylation changes of the 43 most common signaling proteins (using a Proteome profiler Kinase assay). SK-N-DZ cells were treated with 60 μM APR-246 for 30 min and 6 h. **(B)** Raw images of the Protein profiler Kinase assays for the four most deregulated proteins and their corresponding normalized fold-change values (SK-N-DZ). **(C)** Western blot analyses of Phospho-ERK1/2 after 0.5 h, 1 h, 2 h, 4 h, and 6 h treatment with 60 μM APR-246 in BE-2C, SK-N-DZ, and NBL-S cells.

To investigate APR-246’s impact on the common cellular signaling pathways, we used the Human Phospho-Kinase Array Kit to measure the phosphorylated fraction of 43 signaling proteins after their exposure to APR-246 at concentration ensuring complete cell death. Results demonstrated a more than 1.5-fold increase in phosphorylated ERK1/2, AKT1/2/3, and total HSP60 and a borderline 1.47-fold increase in c-Jun (**Figures 1A, B**). Phosphorylated CREB was the only protein that decreased more than 1.5-fold (**Figure 1A**). The results for phosphorylated ERK1/2 contrasted with a previous publication on colorectal cancer cell lines where APR-246 was described as an MEK1/2 inhibitor (23). ERK1/2 is an end-point kinase of the RAS-MAPK pathway, one of the most commonly dysregulated pathways in NB (24). The RAS-MAPK pathway’s signaling branches into PI3K/mTOR/AKT signaling (24) and, thus, an increase in phosphorylated AKT1/2/3 is expected as a consequence of an activated RAS-MAPK pathway. To understand whether the dynamics of EKR1/2 phosphorylation could explain the discrepancy between our observations and previous ones, we investigated ERK1/2 protein expression over time (**Figure 1C**: Western blot analyses at 30 min, 1 h, 2 h, 4 h, and 6 h after APR-246 exposure). Results demonstrated that APR-246 was capable of inhibiting ERK1/2 phosphorylation in BE-2C and SK-N-DZ cells shortly after their exposure to it, but after 3 - 6 h ERK1/2 phosphorylation increased. Measurements at 6 h were following observations obtained using the Human Phospho-Kinase Kit at the same time point, validating the results (**Figure 1C**).

### ERK1/2 affects cell survival after APR-246 treatment by modulating apoptosis

**Figure 2:**
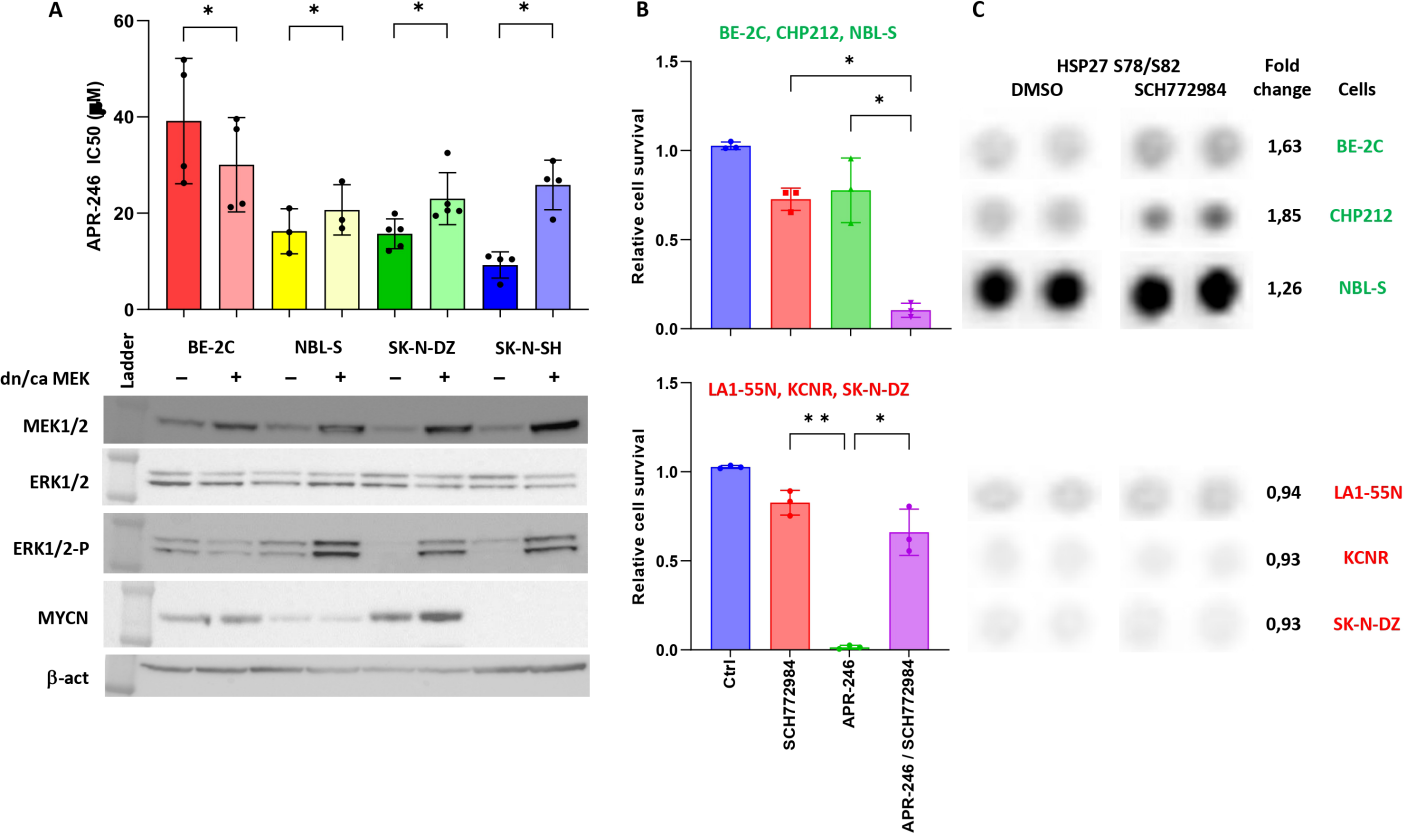
ERK1/2 signaling affects cell resistance to APR-246. **(A)** The IC_50_ of cells transfected with either pmRNA5-dnMEK (BE-2C) or pmRNA5-caMEK (NBL-S, SK-N-DZ, and SK-N-SH). Significant decrease (BE-2C: 1.30-fold, p=0.015) and increases (NBL-S: 1.27, p=0.043; SK-N-DZ: 1.46, p=0.043; SK-N-SH: 2.80-fold, p<0.01) in IC_50_ were observed in cells with lower or higher phospho-ERK1/2 levels, respectively. Corresponding Western blot analyses after stable transfection of pmRNA5-caMEK or pmRNA5-dnMEK into BE-2C, NBL-S, SK-N-DZ and SK-N-SH cells. The transfection caused significant upregulation of MEK1/2 and phospho-ERK1/2 when using pmRNA5-caMEK (NBL-S, SK-N-DZ, and SK-N-SH) and slight inhibition of phospho-ERK1/2 when using pmRNA5-dnMEK (BE-2C). **(B)** Interactions of ERK1/2 inhibitor (SCH772984) with APR-246. We were able to stratify cells into groups showing strong synergies (graph above: BE-2C, CHP212, and NBLS-S) and strong antagonism (graph below: LA1-55N, KCNR, SK-N-DZ) between SCH772984 and APR-246. The following concentration of APR-246 and SCH772984 were used respectively: BE-2C (30 μM; 5 μM), CHP212 (20 μM; 1 μM), NBL-S (20 μM; 10 μM), LA1-55N (30 μM; 10 μM), KCNR (30 μM; 10 μM), and SK-N-DZ (30 μM; 10 μM). Every point represents one cell line. **(C)** Raw images of the Proteome profile Kinase assay for phospho-Hsp27 in BE-2C, CHP212, NBL-S, LA1-55N, KCNR, and SK-N-DZ cells following 6 h of SCH772984 pretreatment. Results show generally higher expression of phospho-Hsp27 at baseline and the significantly higher induction (1.69-fold, p=0.019) of phospho-Hsp27 after 6 h of SCH772984 pretreatment (with the indicated concentrations) in BE-2C, CHP212, and NBL-S cells. Concentrations of SCH772984 were the same as in A.

**Figure 3:**
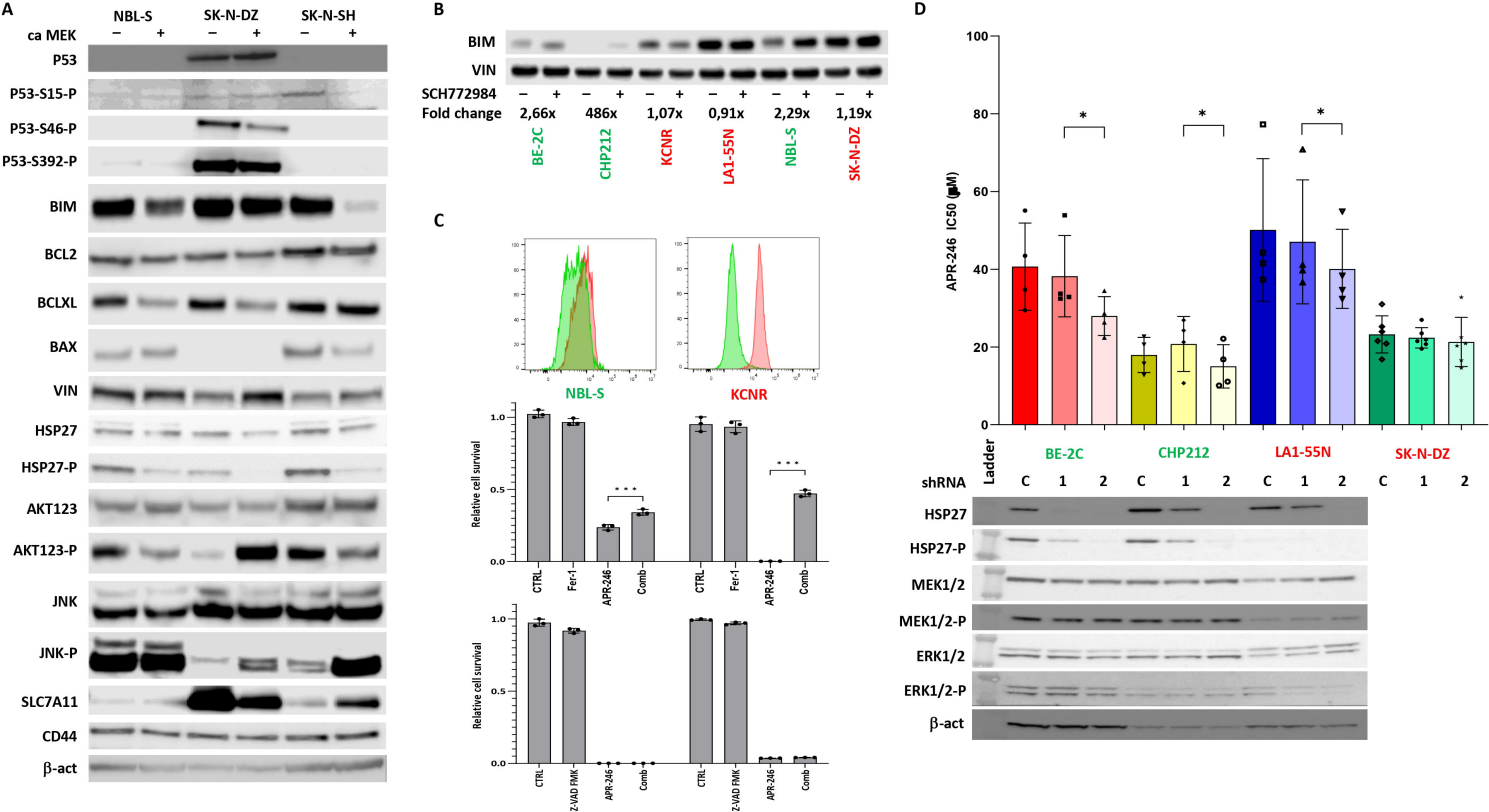
Effects of ERK1/2 signaling on components of apoptotic pathway. **(A)** Western blot of components of apoptotic pathway and AKT123 and JNK signaling. The results demonstrate that activation of ERK1/2 decreased the BIM expression and AKT phosphorylation in NBL-S and SK-N-SH cells but had no effect on BIM expression and increased AKT phosphorylation in SK-N-DZ cells. Phospho-Hsp27 levels were decreased in NBL-S, SK-N-DZ, and SK-N-SH cells. **(B)** Increase in levels of pro-apoptotic BIM after SCH772984 pretreatment in BE-2C, CHP212, and NBL-S cells. BIM levels remained unchanged in KCNR, LA1-55N, and SK-N-DZ cells pretreated with SCH772984. Concentrations of SCH772984 were the same as in **(A)**. **(C)** Detection of lipid peroxidization using Image-iT™ Lipid Peroxidation Kit. KCNR cells demonstrated higher activation of ferroptosis than NBL-S cells. Ferrostatin-1 (Fer-1) (5 μM) but not Z-VAD-FMK (50 μM) was able to rescue KCNR and NBL-S cells from APR-246-induced (25 μM) cell death. **(D)** Knock-down Hsp27 had a significant impact on APR-246’s IC_50_ decrease in BE-2C (1.46-fold, p=0.03), CHP212 (1.25-fold, p=0.04), and LA1-55N (1.19-fold, p=0.04) cells. There was no impact on APR-246’s IC_50_ in Hsp27 knock-down SK-N-DZ cells that did not express it and were used as a negative control. Western blot analyses show that Hsp27 shRNA1 partially blocks the expression of Hsp27, and shRNA2 completely blocks it, as seen in the BE-2C, CHP212, and LA1-55N cells that express Hsp27. Silencing Hsp27 had no impacts on the components of the RAS-MAPK signaling pathway, MEK1/2, and ERK1/2. *<0.05, ***<0.01.

To understand how ERK1/2 is involved in APR-246-induced cell death, we modulated the phosphorylation of ERK1/2 by introducing a constitutive active-MEK (caMEK) into NBL-S, SK-N-DZ, and SK-N-SH cells that demonstrate low levels of phospho-ERK1/2 and a dominant negative-MEK (dnMEK) into BE-2C cells that have the highest levels of phospho-ERK1/2 (**Figure 2A**, lanes for MEK1/2, ERK1/2, and ERK1/2-P). The results demonstrated that the introduction of caMEK into NBL-S (1.27-fold), SK-N-DZ (1.46-fold), and SK-N-SH (2.80-fold) cells increased the phosphorylation of ERK1/2, and the IC_50_ of APR-246 (**Figure 2A**). In contrast, the introduction of dnMEK decreased (1.30-fold) the IC_50_ of APR-246 in BE-2C cells (**Figure 2A**). Increased levels of phosphorylated ERK1/2 did not affect ERK1/2, MYCN, or p53 (**Figures 2A**, **3A**). A decrease in phosphorylated p53 after ERK1/2 phosphorylation was observed in SK-N-SH cells at S15 and in SK-N-DZ at S46 (**Figure 3A**).

Because phosphorylation of the ERK1/2 affected APR-246 efficacy, we investigated possible interactions between APR-246 and inhibitors of the ERK1/2 and RAS-MAPK pathway. We used SCH772984, an inhibitor of the ERK1/2 RAS-MAPK end-point kinase, and alectinib, a second-generation ALK inhibitor, to inhibit the RAS-MAPK pathway at its end and beginning, respectively.

APR-246 and SCH772984 co-treatment showed a mixed response. We observed synergistic effects in BE-2C, CHP212, and NBL-S cells but a strong antagonistic effect in LA1-55N, KCNR, and SK-N-DZ cells (**Figure 2B**). The latter cells also presented with strong antagonism when using APR-246 in association with alectinib (**Supplementary Figure S1**). In contrast, NBL-S cells showed synergistic effects (**Supplementary Figure S1**), and BE-2C and CHP212 cells only showed an additive effect (**Supplementary Figure S1**).

To understand whether short- or long-term cellular exposure was responsible for changes in APR-246’s IC_50_, we investigated the antagonistic effects of SCH772984 on LA1-55N cells at 0 h, 1 h, 3 h, 6 h, and 12 h before APR-246 exposure. Results demonstrated that immediate or 1 h prior exposure to SCH772984 did not induce resistance to APR-246. The first effect was visible after 3 h of exposure, and resistance was strongest at 6 h and 12 h (**Supplementary Figure S2**).

### Effects of ERK1/2 signaling on apoptosis

To identify the possible origin of the synergistic and antagonistic effects of ERK1/2 inhibitor SCH772984 on cellular response to the APR-246, a proteome profiler was used to identify the activation of signaling kinases after treatment with SCH772984. We identified increased Hsp27 phosphorylation after SCH772984 treatment as a marker of increased efficacy of APR-246 (**Figure 2C**). This result suggested that ERK1/2 could affect components of the apoptotic pathway differently in synergistic and antagonistic cell lines. Using WB, we investigated the expression of main apoptotic proteins in caMEK NB cells. The results demonstrated that BIM was decreased in two synergistic cell lines NBL-S and SK-N-SH after ERK1/2 activation while it showed no change in antagonistic cell line SK-N-DZ (**Figure 3A**). All other important components of the apoptotic pathway, BCL2, BCLXL, BAX, and Hsp27 pathways did not show any changes consistent with synergistic or antagonistic behavior (**Figure 3A**). Interestingly phosphorylated Hsp27 demonstrated a decrease in all three cell lines after ERK1/2 activation while changes in BCLXL and BAX were cell line specific. In addition to that we investigated activation of two important signaling pathways AKT123 and JNK. AKT123 showed a decrease in phosphorylation in NBL-S and SK-N-SH cells and an increase in phosphorylation in SK-N-DZ after ERK1/2 inhibition. JNK phosphorylation increased in SK-N-DZ and SK-N-SH but it did not correlate with the antagonistic or synergistic response of the cells (**Figure 3A**). CD44, an important component of the xCT transporter (also known as SLC7A11) did not show any changes after ERK1/2 activation. SLC7A11 showed upregulation in SK-N-SH cells and slight downregulation in SK-N-DZ cells where it was highly expressed (**Figure 3A**).

Since one of the main targets of phospho-ERK1/2 is pro-apoptotic BIM (24) and due to the above results, we investigated BIM expression after SCH772984 treatment. WB showed that BIM increases after SCH772984 treatment in cells with synergistic behavior but there was no change in BIM after SCH772984 treatment in cells that demonstrated an antagonistic relationship between APR-246 and SCH772984 (**Figure 3B**). In our previous publication we showed that despite the apparent activation of apoptosis as measured by Cas3/7 activity, the caspase inhibitors could not stop the apoptosis (8). To understand if antagonistic cell lines could undergo ferroptosis we compared NBL-S and KCNR cell lines for the presence of lipid peroxidation, a biomarker of ferroptosis. The result showed strong and weak induction of lipid peroxides in KCNR and NBL-S respectively after treatment with APR-246 (**Figure 3C**). Co-treatment with ferrostatin-1 was able to rescue KCNR cells and to a lesser extent NBL-S cells, demonstrating that activation of ferroptosis is more pronounced in KCNR than NBL-S cells. Similar to our previous publication (8) pan-caspase inhibitor Z-VAD-FMK could not rescue both cell lines from APR-246-induced cell death.

Next, we also clarified Hsp27’s involvement in cell sensitivity to APR-246. We created four Hsp27 knock-down NB cell lines with high baseline Hsp27: BE-2C, LA1-55N, CHP212 (**Figure 3D**), and SK-N-DZ as negative control (no Hsp27 expression, not shown here). Total Hsp27 knock-down significantly decreased APR-246’s IC_50_ (**Figure 3D**). Hsp27 knock-down showed no effects on non-phosphorylated and phosphorylated MEK1/2 and ERK1/2 (**Figure 3C**), p53, and MYCN (**Supplementary Figure S3**) but it decreased phosphorylated p53 as evaluated using western blot analysis (**Supplementary Figure S3**). No changes in internal GSH levels were observed (**Supplementary Figure S4**).

### MEK1/2 and ERK1/2 modulate cysteine uptake and SLC7A11 phosphorylation

**Figure 4:**
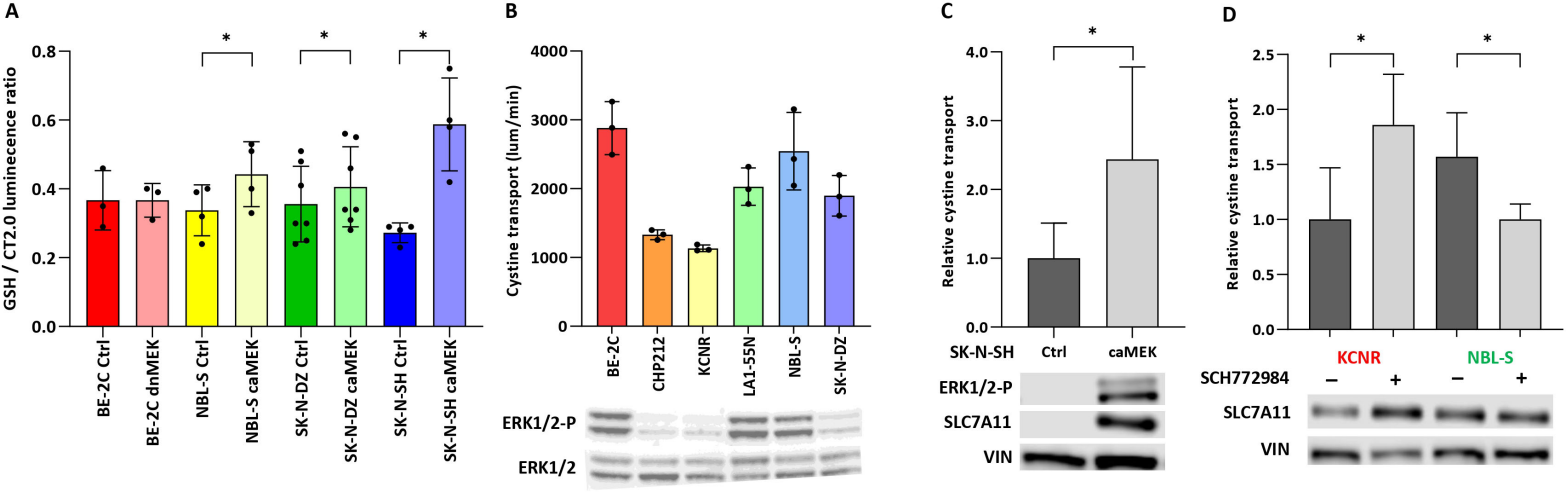
Effect of ERK1/2 signaling on glutathione and cystine transport. **(A)** Intracellular concentrations of GSH after ERK1/2 activation. Significant 1,3.-fold (p<0.01), 1,11-fold (p=0.04) and 2,19-fold (p=0.02) differences in GSH levels were observed in NBL-S, SK-N-DZ, and SK-N-SH cells, respectively. **(B)** Correlation of baseline cystine-FITC transport in the presence of phospho-ERK1/2 demonstrated that cells with higher levels of phospho-ERK1/2 exhibited higher baseline cystine transport. **(C)** Cystine transport, measured by cystine-FITC in SK-N-SH pmRNA5-ctrl or pmRNA5-caMEK cells, demonstrated a 2.44-fold (p=0.042) increase. Western blot demonstrates an increase in ERK1/2 and SLC7A11 phosphorylation in caMEK SK-N-SH cells. **(D)** Cystine transport, measured by cystine-FITC, increased 1.86-fold (p=0.037) and decreased 1.57-fold (p=0.042) in KCNR and NBL-S cells, respectively, after 6 h of pretreatment with 10 μM SCH772984. Western blot shows the respective increase and decrease in SLC7A11 phosphorylation after pretreatment with 10 μM SCH772984. *<0.05.

Our group and others recently demonstrated that APR-246 is detoxified by GSH and molecules possessing free -SH groups (8, 11). We therefore investigate how phosphorylation of ERK1/2 might impact intracellular GSH levels. Intracellular GSH showed a significant increase in NBL-S (1.3-fold), SK-N-DZ (1.11-fold), and SK-N-SH (2.19-fold) caMEK cells in comparison to controls (**Figure 4A**). The expression of SLC7A11 (also known as xCT) was established as a major marker of sensitivity to APR-246 because it transports cystine, a precursor of cysteine that has a free -SH group (12). Measuring baseline cystine-FITC uptake in NB cells suggested that cells with higher phosphorylated ERK1/2 levels might transport cystine-FITC faster than cells with lower baseline pERK1/2 (**Figure 4B**). SLC7A11 is a target of the PI3K-mTOR-AKT axis (25) that is known to cross-talk with the RAS-MAPK pathway (**Figure 3A**). To investigate whether RAS-MAPK pathway modulation might affect SLC7A11 activity, we tested the ability of caMEK SK-N-SH cells (**Figure 4C**), and of NBL-S and KCNR cells pretreated with SCH772984 (**Figure 4D**) to take up cystine-FITC. Results showed that caMEK SK-N-SH cells transported cystine-FITC almost 2.5-fold faster than control SK-N-SH cells (**Figure 4C**). Pretreated KCNR and NBL-S cells transported cystine-FITC 2-fold faster and 1.6-fold slower than controls, respectively (**Figure 4D**). Western blot demonstrated that increased phosphorylation of ERK1/2 (**Figure 4C**) and the pretreatment with SCH772984 (**Figure 4D**) affected the total SLC7A11 expression in SK-N-SH and KCNR cells, respectively. SLC7A11 did not show any phosphorylation after enriching the total protein extract for a phosphorylated fraction (result not shown). Finally, no significant changes in intracellular levels of GSH were detected after 6 h of SCH772984 treatment suggesting that the effects of ERK1/2 inhibition are due to the cystine transport and not GSH levels (**Supplementary Figure S5**).

### Erastin and sulfasalazine inhibit the antagonistic effects of ERK1/2 inhibition by SCH772984

**Figure 5:**
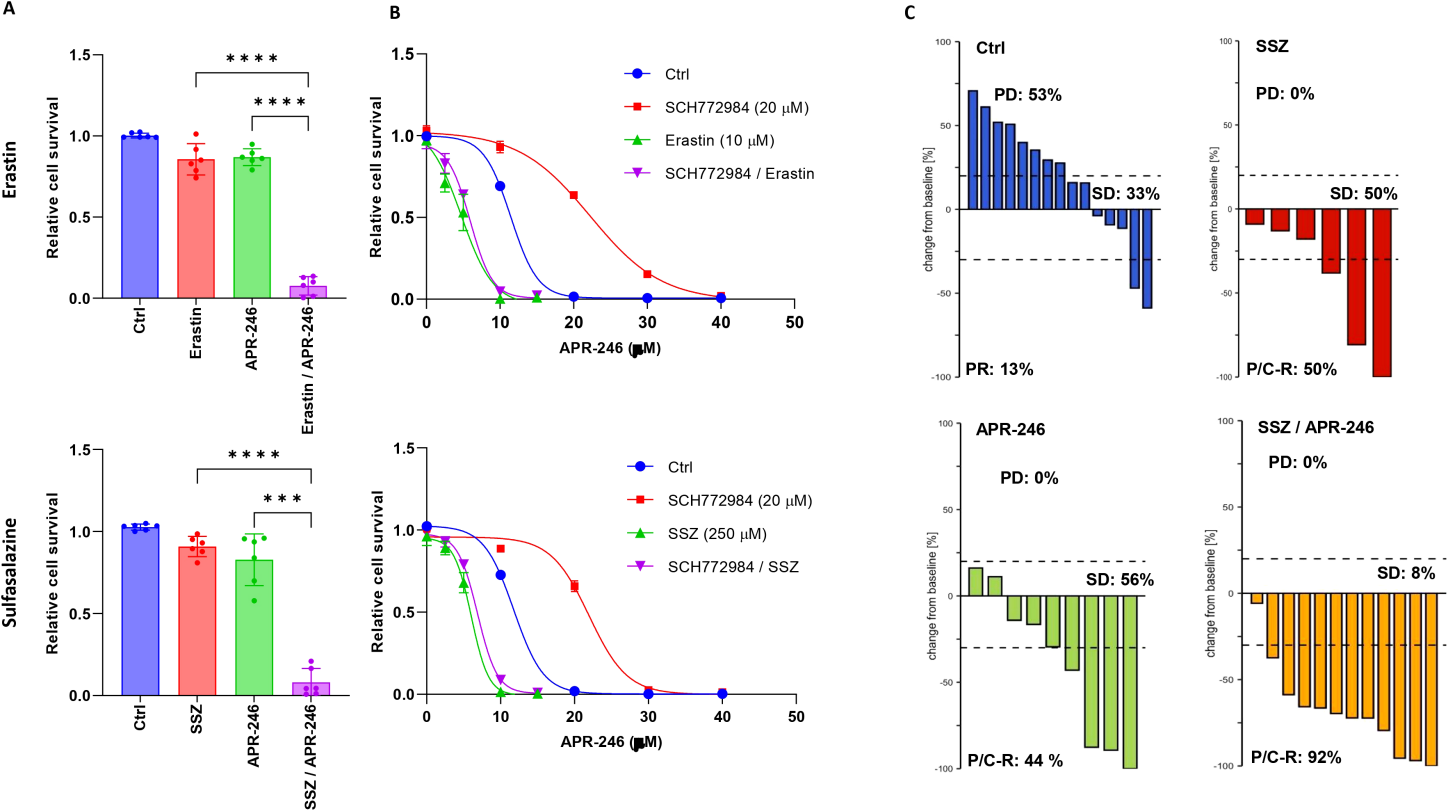
Synergistic actions of SLC7A11 inhibitors erastin and SSZ with APR-246 *in vitro* and *in vivo*. **(A)** Interactions of APR-246 with erastin or SSZ. Results showed strong synergies between APR-246 and erastin or SSZ in the six cell lines. The following APR-246, erastin and SSZ concentrations were used respectively: BE-2C (30 μM, 10 μM, 250 μM), CHP212 (30 μM, 10 μM, 250 μM), NBL-S (10 μM, 10 μM, 250 μM), KCNR (10 μM, 10 μM, 250 μM), LA1-55N (10 μM, 75 nM, 50 μM), SK-N-DZ (10 μM, 75 nM, 150 μM). Every point represents one cell line. **(B)** Erastin and SSZ can reverse the antagonistic effects of SCH772984 in KCNR cell line. **(C)** Waterfall plots demonstrating changes in tumor volume for the 15 IMR-32 DMSO treated xenografts (blue), the 6 IMR-32 SSZ (250 μM) treated xenografts (red), the 9 IMR-32 APR-246 (50 μM) treated xenografts (green) and the 12 IMR-32 xenografts (yellow) treated with combination of SSZ (250 μM) and APR-246 (50 μM). Results demonstrated substantially higher rates of partial or complete response (92%) in the combination group (yellow) than in the single SSZ (P/C-R: 50%) or APR-246 (P/C-R: 44%) treatment groups.

Erastin and SSZ were previously described as potent inducers of ferroptosis and inhibitors of SLC7A11 cystine transport (12, 26, 27). Co-treatment experiments with APR-246 demonstrated that there was a very good synergy between these compounds in six NB cell lines (**Figure 5A**). To understand whether erastin and SSZ could reverse the antagonism induced by SCH772984, co-treatments of erastin or SSZ with SCH772984 and APR-246 were performed. Both erastin and SSZ were able to fully reverse the antagonistic effects of SCH772984 in KCNR cells (**Figure 5B**).

### *In vivo* efficacy of APR-246 and sulfasalazine cotreatment in NB xenografts

Finally, to investigate the feasibility of combining APR-246 and SSZ *in vivo*, we tested these compounds individually and together in a previously described IMR-32 cell line xenograft-zebrafish model (9). Results showed that IMR-32 cells proliferated in this model with 53% of xenografts showing progressive disease (PD) in the non-treatment control group. Treatment with either 250 μM SSZ or 50 μM APR-246 resulted in complete inhibition of PD and partial or complete remission (P/C-R) of 50% and 44% respectively. Combined treatment resulted in 92% P/C-R, demonstrating a better-than-expected simple additive effect (78%) (**Figure 5C**).

## Discussion

APR-246 was initially described as a compound able to reconstitute the activity of mutated p53 (10, 11). A significant number of studies demonstrated that APR-246 efficiently eradicated cancer cells in the absence of p53 (16). Several studies have demonstrated that p53 has an indirect effect on cellular survival, involving several different molecular pathways in response to APR-246 treatment (8, 16). Using the molecular screening of common cellular signaling pathways, we identified the RAS-MAPK pathway as the most responsive after APR-246 treatment. When investigating its involvement in response to APR-246, we demonstrated that cells with over-activated ERK1/2 were more resistant to APR-246. The increase in resistance comes most likely from an increase in the intracellular concentration of GSH and faster cystine transport through the SLC7A11 antiporter.

The RAS-MAPK pathway is currently a high-priority target for pharmacological intervention because it is one of the most frequently deregulated pathways in cancers (24). Our APR-246 co-treatment experiments with up-stream (alectinib) and down-stream (SCH772984) inhibitors of the RAS-MAPK pathway yielded conflicting results. Strong synergies were observed in some cell lines, whereas strong antagonistic effects were observed in others. These differences in response came from difference in the ability to transport cystine into cells. Although the increased speed of cystine acquisition did not result in increased concentrations of GSH, our previous work demonstrated that precursors of GSH possessing free -SH groups, such as N-acetyl-cysteine, effectively protected NB against cell death induced by APR-246 (8). Notably, the increase in cystine transport was associated with the increase in SLC7A11 expression either by increasing ERK1/2 phosphorylation (SK-N-SH) or treatment with SCH772984 (KCNR). We didn’t observe a decrease in SLC7A11 in NBL-S cells after SCH772984. Cystine is transported into the cells also by X_AG_ sodium-dependent transporters. That transport system might be important for NBL-S cells given their relatively low expression of SLC7A11 (28). Therefore the decrease of cystine transport into the cells after inhibition of ERK1/2 with SCH772984 may be associated with the X_AG_ system. Interestingly, we did not observe that SLC7A11 is regulated by phosphorylation as in other cell types (25).

The present study also identified the involvement of Hsp27 and BIM in the cellular response to APR-246. Both molecules are modulated by ERK1/2 (24) and are also associated with apoptotic and ferroptotic cell death (29–31). Our results demonstrated that activation of ERK1/2 decreased Hsp27 phosphorylation. This result is consistent with studies demonstrating that dephosphorylated Hsp27 forms longer tubular structures involved in cells’ general resistance to multiple different chemotherapeutic agents (32). Pro-apoptotic BIM, on the other hand, was upregulated in cell lines demonstrating a synergistic response to APR-246 and SCH772984 co-treatment. Both BIM and phosphor-Hsp27 remained unchanged in antagonistic cells suggesting an opposite effect of SCH772984 on the response to APR-246. Thus, the phosphorylation status of SLC7A11 and Hsp27, or levels of BIM after treatment with SCH772984 could serve as a potential biomarker for the evaluation of drug-drug interaction of the RAS-MAPK pathway inhibitor and APR-246.

Previous studies have demonstrated that erastin and SSZ are potent activators of cellular death in NB. This effect is brought about, at least in part, by the inhibition of SLC7A11, although other mechanisms, such as the induction of ferroptosis, cannot be excluded (33–35). In the present study, we replicated the excellent synergistic effects between erastin or SSZ and APR-246, as demonstrated previously (12, 13). Importantly, both reagents could fully reverse the antagonistic effects of the SCH772984 ERK1/2 inhibitor. Finally, to assess the translatability of the SSZ and APR-246 combination, we performed *in vivo* experiments on zebrafish embryos xenografted with IMR-32 cells. These experiments demonstrated the synergistic behavior of SSZ and APR-246, suggesting that a combination of these drugs could be deployed rapidly in clinical practice, given that SSZ is already in clinical use and APR-246 passed phase I/II toxicological studies.

## Conclusions

APR-246 has good potential for clinical use against NB, but the exact mechanisms of its actions remain elusive. The present work demonstrated that the RAS-MAPK pathway is an important modulator of cellular response to APR-246 via the modulation of cystine uptake and a subsequent increase in GSH. Cystine transport is regulated on the level of SLC7A11 phosphorylation and not on the level of its expression. Finally, we demonstrated that a combination of APR-246 and RAS-MAPK pathway inhibitors could produce both synergistic and antagonistic effects. Nevertheless, by using erastin or SSZ, we were able to overcome this antagonistic behavior, thus demonstrating the excellent potential for translatability of the SSZ-APR-246 pairing alone or in combination with a RAS-MAPK pathway inhibitor.

## Data Availability

The raw data supporting the conclusions of this article will be made available by the authors, without undue reservation.

## Supplementary material

Supplementary Figure 1 Interaction of Alectinib and APR-246.

Supplementary Figure 2 Effects of SCH772984 pretreatment duration on its antagonistic efficacy against APR-246. LA1-55N cells were pretreated for 0 h, 1 h, 3 h, 6 h, and 12 h with SCH772984 (10 μM). Results show that at least 3 h of pretreatment was needed to produce an antagonistic effect in LA1-55N cells.

Supplementary Figure 3 Effect of Hsp27 on MYCN, p53 and phopho-p53. Hsp27 has no impact on the levels of MYCN, and p53 but is associated with lower levels of phospho-p53 in BE-2C cells.

Supplementary Figure 4 Effect of Hsp27 on GSH. Hsp27 did not affect intracellular concentrations of GSH (p>0.05 in BE-2C, CHP212, LA1-55N, SK-N-DZ).

Supplementary Figure 5 Expression of SLC7A11 in after inhibition of ERK1/2 with SCH772984. No impact of 6 h pretreatment with SCH772984 on total SLC7A11. Cells were treated with following concentrations of SCH772984: BE-2C (5 μM), CHP212 (1 μM), NBL-S (10 μM), LA1-55N (10 μM), KCNR (10 μM), and SK-N-DZ (10 μM).

Supplementary Figure 6 Effects of SCH772984 on levels of intracellular GSH. SCH772984 had no significant effects (p>0.05 in all cell lines) on intracellular GSH levels in the set of six NB cell lines (BE-2C (5 μM), CHP212 (1 μM), NBL-S (10 μM), LA1-55N (10 μM), KCNR (10 μM), and SK-N-DZ (10 μM)).
